# Pregnant women’s experiences with a pelvic floor muscle training program in Nepal

**DOI:** 10.1080/16549716.2021.1940762

**Published:** 2021-08-12

**Authors:** Ann-Katrin Stensdotter, Anette Håland, Borgunn Ytterhus, Satya Shrestha, Britt Stuge

**Affiliations:** a Faculty of Medicine and Health Sciences, Department of Neuromedicine and Movement Science Norwegian University of Science and Technology, NTNU, Trondheim, Norway; b Department of Public Health and Nursing, Science, Faculty of Medicine and Health Sciences, The Norwegian University of Science and Technology, NTNU, Trondheim, Norway; c Department of Nursing, Kathmandu University School of Medical Sciences, Kathmandu University Dhulikhel Hospital, Dhulikhel, Nepal; d Division of Orthopaedic Surgery, Oslo University Hospital, Oslo, Norway

**Keywords:** Self-care, uterine prolapse, urinary incontinence, exercise, compliance

## Abstract

**Background:**

In Nepal, pelvic floor disorders affect about 24% of the women in reproductive age whereof 10% suffer from pelvic organ prolapse (POP). Still, many do not seek health care. Strengthening exercises for the pelvic floor muscles for prevention and treatment of POP has shown strong evidence internationally, but for women in Nepal surgery is primarily offered. To amend this, a novel pelvic floor muscle training (PFMT) program for pregnant women was introduced.

**Objective:**

To learn about how the PFMT-program was received by the participating women, their understanding of the importance of doing the exercises, and the constraints of daily life for performing the program.

**Methods:**

A qualitative study design based on a sub-sample (N = 10) from a strategic sample (N = 235) who participated in the PFMT-program. Ten semi-structured in-depth interviews were interpreted according to a phenomenological analytical tradition.

**Results:**

The 10 women were representative for the women who had participated in the PFMT-program with regard to urban residence, socioeconomic, and educational standing. The program was well received and compliance satisfactory. In line with the PFMT’s learning outcomes, the women described risk factors, showed knowledge about the pelvic floor muscles, and understood the importance of doing the exercises. They had managed to fit the exercises into their busy daily routines. Meeting peers in exercise groups and understanding from family were positive factors for compliance.

**Conclusion:**

The Nepalese women appear interested in self-care and are making an effort to fit the exercises into their busy schedule. Although the communicative validity was satisfactory, the pragmatic validity cannot be generalized to women in rural areas and under less fortunate socioeconomic and educational circumstances.

## Background

Pelvic floor disorders affect approximately 28 million women worldwide, comprising several conditions such as urine incontinence (UI), pelvic organ prolapse (POP), and uterine prolapse (UP) [1], affecting particularly women who have given birth [2]. POP is characterized by the uterus descending into or beyond the vagina. The condition is graded from 0 (no problem) to 4 (total prolapse) [3]. Approximately 60% suffering POP also present with UI [4]. The global incidence is estimated to nearly double in the next 40 years due to increased life expectancy [5], as POP is more frequent in postmenopausal women [6].

The heaviest burden fall on low-income countries such as Nepal [7], where complications in pregnancy and childbirth are major causes of morbidity and mortality [8]. Although maternal mortality has declined ~ 30% during the last 20 years [9], morbidity such as POP and UI remain high, particularly in the rural areas, at 8% and 24% respectively [10]. According to the literature, prevalence of POP in Nepal varies widely between districts (2.8–44.5%) [11]. Of the sufferers, estimated 33% need gynecological surgery, being the second most common operation performed in Kathmandu hospitals [8]. Precise estimation of the number of women suffering pelvic floor disorders is challenging as statistics typically include only cases treated surgically [12]. Sometimes unable to identify the symptoms as unnormal, women fail seeking treatment before the condition has become severe [13].

A cardinal symptom of POP is the feeling or appearance of a vaginal bulge. Additional symptoms can be urinary- or bowel related, or present difficulties in sexual activity [14]. In Nepalese women this was reported by 68%, 42%, and 73.9%, respectively [15]. Because of these limitations many endure humiliation, harassment, and torment from husband and family members. Sometimes the husband leaves the wife [15]. Risk factors for POP are strenuous physical work during and immediately after pregnancy, lack of skilled birth attendants, malnutrition, high parity, early marriage, smoking, chronic obstructive pulmonary disease (COPD) [16], and woodsmoke from cooking [17]. The latter causing strain on the pelvic floor from incessant coughing [16] and oxidative stress on the tissues [18]. Furthermore, Nepalese women are small, averaging 150 cm [19], while birth weight averages 3000 g [20]. POP is still stigmatized in Nepal and most women need approval from their spouse or mother-in-law to seek healthcare [21]. Many feel shame or may think the condition is normal for child bearing women [15].

The Ministry of Health and Population of Nepal has taken the problem seriously and dedicated a part of the budget in 2008 to finance free surgery for women suffering from POP [22]. Successful surgery has improved women`s experience of quality of life by reducing both physiological and psychological suffering [23]. Surgical camps in Nepal have however received criticism related to lack of postoperative follow-up and limited available resources for providing quality health services which have resulted in health complications [23–25]. An additional problem post surgically is the common lack of access to adequate hygiene facilities in the home setting c.f [26].

By providing surgery as the sole solution, the government initiatives have overlooked the opportunity to treat women in the early stage. The primary solution to POP internationally is conservative, using pessaries [27]. This is however problematic in Nepal due to limited health services and hygiene facilities. A non-invasive solution not encountering these problems is pelvic floor muscle training (PFMT), internationally recommended as first-line treatment, shown to prevent occurrence and reduce progression and symptoms associated with POP [28,29] and UI without adverse effects [30,31]. Another advantage of PFMT is that it does not require expensive resources and can reach women also in rural and remote areas [32]. Despite recommendations, PFMT has however not been commonly practiced or studied in Nepal [27]. Brief verbal instructions combined with an illustrated leaflet has alone proven insufficient to make Nepalese women perform the exercises correctly [33]. In contrast, a recent feasibility study including video and supervised exercises showed that pregnant women were able to perform PFMT both at the hospital and at home [34]. The study did however not include any qualitative information about the women’s experience with the PFMT-program.

To enhance compliance with and retention of proper performance of the exercises, it is important to understand aspects of motivation and barriers from the perspective of the participating women. This is particularly important as the PFMT program, albeit adapted to Nepali culture, originated from the Western culture.

In this study we therefore interviewed a sub-sample of the women who participated in the above mentioned PFMT program to learn about how it was perceived and understood; particularly their understanding of the importance of doing the exercises and the constraints of daily life for performing the program. The rationale for this study was to gain feedback information that can be used to improve future programs.

## Method

### Design and epistemology

This qualitative work was based on interpretive phenomenological analytical tradition [35], considered appropriate for accessing the Nepalese women’s conscious descriptions of their experiences of participating in the PFMT-program during pregnancy, told in retrospect after having given birth. Their experiences were revealed by using semi-structured individual qualitative interviews. Idealistically according to this method, an open interview would have been preferred. But due to the authors’ local knowledge on the Nepali socio-cultural context and existing research findings revealing the stigma of talking about POP [36], we considered semi-structured interviews best suited.

### Setting and research population

This article reports on qualitative data from a sub-sample (n = 10) recruited post-partum among participants in a strategic sample of 253 women who had completed a PFMT-program for pregnant women at the perinatal clinic at Dhulikhel Hospital. The recruitment of the sub-sample was practically organized by the perinatal clinic at the hospital. An effort was made to include women with diverse socio-economic background. To be eligible for participation, the women had to be able to speak and understand Nepali. Women with known history of mental illness were excluded. The recruitment was continued until empirical saturation of information was satisfied. Interviews were carried out during September – December 2018. The PFMT-program was organized in collaboration between Dhulikhel Hospital, Kathmandu University School of Medical Science and Oslo University Hospital, Norway.

### The PFMT-program

The program, originating from the Western culture, was adapted to the Nepali context, and consisted of attending a minimum of four follow-up visits to the clinic for education and supervised PFMT, and daily home exercises during pregnancy. The women were individually taught how to control the pelvic floor muscles using biofeedback by a vaginal probe to monitor contraction and relaxation. The educational material consisted of a Nepal-produced instructional video (https://youtu.be/XsDpfq10JMI), a leaflet, and an exercise diary. See Acharya et al. for details [37]. Several women brought their husband, sister, or another family member to the hospital for the first antenatal control and the first PFMT-visit.

### Data collection

The data collection was based on semi-structured qualitative interviews as recommended when the ambition is to uncover lived experiences and unfold meaning from the informant’s point of view [38]. The first interview guide was written in English and then translated into Nepali by a native Nepalese (4^th^ author), who also performed the interviews. To make sure the interviews were well prepared, a pilot was carried out on site at the hospital in Nepali to test and improve the content validity of the interview-guide. The precision in a few questions was increased to avoid misunderstanding due to Nepali language ambiguity and cultural understanding. The interviewer was a nurse experienced in the research method. To obtain rich data, two physiotherapists who were involved in the PFMT-program and one professor from the Dept. of Nursing with expertise in qualitative methods were appointed as mentors. Those were present as observers to support the interviewer during the first two interviews and gave feedback afterwards. The following interviews were carried out with only the interviewer and the informant present. All interviews occurred in a private room at the perinatal clinic, except two that were conducted in the home of the informant. An audio device was used to record the interviews. The same person who performed the interviews also transcribed all audio recordings and performed all translations into English.

To meet our objective to learn about how the PFMT-program was perceived and experienced we used a semi-structured interview with five main topics; (i) women’s knowledge prior to the exercise program, (ii) narratives on their experiences of participating in the program, (iii) their performance of the exercises, and (iv) experiences of learning outcome. At the end of the session the women were given an opportunity to talk about matters not covered in the interview.

### Data analysis

We treated the transcribed and translated interviews as one set of data. The first step was to read all the transcripts to get an overview of the total content and to get an impression of what appeared to be of significant relevance to the main objective of the study. The second step was to de-contextualize the total by identifying *meaning-bearing units* in each interview, which were written as close to the women’s own expressions as possible. The third step wrapped up these meaning-units into *central meaning-themes* that were transformed into *analytical themes* in accordance with the study objective, keeping them as simple as possible. This process alternated between the women’s stories and the researchers’ theoretical stance within each separate interview and between the combined interviews as a set. This process is described as ‘ … *thematizing the statements from the subject’s viewpoint as understood by the researcher*’ [38]. The researcher is always aware of the own preconception about the material and makes an effort towards a neutral approach. In the fourth and final step we condensed the themes into categories and turned them into a text based on our interpretations. This made us go beyond the verbatim of the translated transcriptions as our interpretations became recontextualized within the existing base of knowledge. Note that this analysis process was not linear but dynamic, moving in-and-out of the steps above to reveal patterns and to make the final decision on meeting the objective of the study (Table 1).

**Table 1.** Illustration of the three analytical steps after the first step of reading through the ten interviews, exemplified with the first analytical theme/category followed by the next two steps. Note that the English in the first column is the verbatim transcription, translated directly from Nepali

Meaning-bearing units	Central meaning themes	Analytical theme/category
I did not know anything about it (PFM) before (PFMT) (interview 2)	Old women share their experiences and knowledge on gynecological problems with young pregnant women.	Gynecological problems during/after pregnancy belong to women’s social world.
Yes, I have heard from our grandmothers and old people say about UP. I used to wonder, what was UP? (interview 4)		
No, I was unknown about it (PFM) before (interview 8)	The women’s narratives focus on the social and practical consequences of pregnancy and birth.	
I didn’t know about it (PFM) before (interview 9)		
Heavy lifting, uterine infection may lead to UP [told her grandmother] (interview 9)	The pregnant women lack knowledge on muscular- and physiological issues before they attend the PFMT-program.	
She [the grandmother] had a ring to fix it but she still has problem of UI (interview 9)		
I didn’t know about it (PFM) before as it was my first time [being pregnant] (interview 10)		
Heavy lifting might cause it [UP] as my mother-in-law told me about it (interview 10)		
Etc. from in total 10 interviews.		

PFM: Pelvic Floor Muscles. PFMT: Pelvic Floor Muscle Training. UP: Uterine Prolapse. UI: Urine Incontinence.

### Ethics

Ethical approval was granted by the Norwegian Centre of Research Data (NSD, 798,629) and the Institutional Review Committee of Kathmandu University School of Medical Sciences/Dhulikhel Hospital (IRC-KUSMS 104/18). Situational and relational ethical challenges regarding cultures and languages were taken into consideration. Regarding impartiality, all researchers present with the women were females with respect to a gendered society where females are sub-ordinated men. This was particularly important regarding the intimate topic. The interviewer was Nepalese from the same district as the informants, which was important for the cultural and social codex. In a hierarchical system such as in Nepal, the inequality in social status between the participating women and the researchers was difficult to reduce. The participants were assured they were not obligated to answer questions that they felt uncomfortable with. All interviews and data material were kept confidentially, and the study was conducted in line with the Helsinki Declaration. Written and oral information about the project was provided to all participants in Nepali, and written informed consent was obtained and securely stored.

## Results

Data from interviews with 10 women was considered satisfactory to reach empirical saturation of information. The sub-sample was according to demography reasonably representative for the women who participated in the PFMT-program. All the interviewed women had full responsibility for the household and half of them were additionally employed or running a business (Table 2). (In this section all citations are given according to the verbatim transcription, translated directly from Nepali).

**Table 2.** Characteristics of the sub-sample relative to the strategic sample who participated in the PFMT program

Variables	Strategic sample n = 253*	Sub-sample n = 10**
Age, years, mean (SD)	25 (4)	25 (4)
Number of pregnancies, median (IQR)	1 (0–2)	2 (1–2)
Body mass index, kg/m^2^, mean (SD)	23 (4)	-
Area n (%) Urban Rural	Kavre 232 (92) 21 (8)	Kavre 8 (100) 0 (0)
Family n (%) Nuclear Extended	89 (35) 164 (65)	2 (25) 6 (75)
Education, years n (%) No education Primary school (1–5) Secondary (6–12) Bachelor and above (>12)	8 (3) 19 (8) 172 (68) 54 (21)	0 (0) 0 (0) 3 (37) 5 (63)
Monthly income n (%) Yes No	58 (23) 195 (77)	--
Occupation, *n* (%)*** Housewife Business (shop, agriculture, etc) Employed (government, private) Other	251 (99) 13 (5) 45 (17) 7 (2)	8 (100) 4 (50) 4 (50)-

*The strategic sample that participated in the PFMT program, data reused from Acharya [37]

**The sub-sample interviewed in the present paper (Missing data, n = 2)

***Women can belong to more than one category

- no information given

### Gynecological problems during/after pregnancy belong to women’s social world

The participating women’s knowledge prior to the exercise program was based upon oral transfer of information mostly from older women in their family and some from other experienced women, often through eaves dropping, not gained from health professionals. Thus, their narratives consisted of the social consequences and practical problems rather than physiological issues. Almost all the participants spontaneously said they knew little or nothing at all about pelvic floor muscles (PFMs) before participating in the PFMT-program. Despite saying they knew little it however became apparent as the interviews progressed that most of the women had a wider understanding of UP and UI than first expressed. The women`s expressions uncovered both differences and similarities in their understanding and revealed whose stories they trusted.

One woman said that she did not fully understand what UP was until she came to the hospital

Yes, I have heard from our grandmothers and old people say about uterine prolapse. I used to wonder what was uterine prolapse. But after I visited hospital, I knew about it and also about pelvic muscles and about the importance of pelvic floor exercise.

Woman 4.

The women had learned about potential risk factors for developing UP from female family members, and how to take care during pregnancy to avoid developing the condition. Some had also been explained about UI and what dealing with that meant:

I knew that heavy lifting during pregnancy causes uterine prolapse.

Woman 7.

Yes, I had {heard about urine incontinence} … I had heard that we can`t control our urination even when we are talking and sitting too … Heavy lifting might cause it {uterine prolapse} as my mother-in-law told me about it.

Woman 10.

Some women said having learned about UP and UI from old females’ chats in the village and from mothers-in-law, while another woman said she heard about UP from her sister-in-law who had been struggling with UP daily after having given birth:

Yes, she told me certain things. I realised from her that one should take much care during pregnancy.

Woman 1.

A minority knew about the physiological aspects on the pelvic floor. Only two women mentioned having heard about PFMs before attending the PFMT-program. One woman said she learned about the PFMs through personal experience during her first pregnancy:

I had a fear my uterus would move from normal position due to weakness but in second childbirth, after exercise, I don`t have any fear regarding this problem.

Woman 4.

Another woman said her mother taught her about the PFMs and their function, explaining that her mother was a health practitioner. But that she did not want her mother to teach her as she was not interested in performing PFMT before marriage:

Yes, I got to know about it [pelvic floor muscles] while my mother was advising other women in our community to perform it [PFMT] by tightening our pelvic muscles during micturition.

Woman 7.

Most of the women had no prior knowledge about the PFMs and said they learned about it at the hospital when attending the PFMT-program. Reasons for lack of knowledge was not having any other females in the household to talk to and not visiting the hospital for control during the first pregnancy.

### Performance of exercises a desired, but sub-ordinated agenda?

There was an agreement among the women that they were aiming, despite leading a busy life, to perform the exercise several times a day during pregnancy as advised by the PFMT-program. The women expressed different preferences when asked about when they did their exercises, depending on their engagement in daily activities and chores. They agreed that finding a convenient time made it easier to perform the program regularly, but still a busy schedule often made them forget.

There was a consensus on preferring to do the exercises at home. Many were facing difficulties when trying to perform the exercises in other situations, such as during work which they experienced was both challenging and distracting. They felt uncomfortable doing it around other people.

Yes, they won`t know but while we are chatting with each other, I get involved in the conversation only, so cannot concentrate.

Woman 1.

An important element about exercise performance mentioned by several of the women was choosing a good position that was comfortable and easy. Training when sitting was usually said to be a comfortable and favoured position, allowing the exercise to be performed while sitting down cooking (cooking often takes place sitting down on the floor by a low stove). The positions of choice varied however depending on personal preference, opportunities during their everyday lives and bodily changes that came with pregnancy.

My body was big, I had gained lots of weight, I felt it more comfortable in sitting, rather than standing [position].

Woman 4.

Lying down on the side was also viewed as comfortable and easy, and some chose this position frequently when performing the exercises.

Yes, I prefer other positions like sleeping position, I find it easy and comfortable.

Woman 4.

### Family support influence the women’s motivation and compliance with exercise performance

An important factor brought up in the interviews about what facilitated motivation and compliance with the PFMT-program was family support. Some women brought someone to accompany them to the hospital appointments or included their families in the process of participating in the PFMT-program. Some women found it easy to share with their husband, saying he was particularly important reminding them to perform the exercises and by showing a positive attitude towards the PFMT-program. Others found sharing challenging; one said her husband often had to stay away from home due to work and the distance between them made sharing troublesome. Instead, she had confided in her mother-in-law who expressed support towards her attendance:

… she [mother-in-law] suggested to me to do if I was advised from the hospital.

Woman 5.

Prominent factors for the women`s inner motivation to perform the PFMT was rooted in their own knowledge of the preventative effect of the exercises, the understanding of the benefits the on their bodies, and the positive feeling after having performed the exercises. One woman said that she first realized how important exercise was after having been told by health professionals at the hospital. Even though her mother used to teach other women about the exercises, it was not until she had interacted with people at the hospital that she understood that the PFMT was relevant also for her. Another woman expressed doing it because it was good for her regardless of finding the exercises difficult to perform:

I used to perform early in the morning … It`s not boring but it was quite difficult due to my large abdomen. I felt uncomfortable and uneasy. I was advised that it will be fruitful for me till I grow old.

Woman 6.

Another important factor for motivation to adhere to the PFMT-program was having a close friend or family member suffering from problems related to the pelvic floor. Seeing with own eyes the implications the condition could have on a person`s life increased the incentive for the women to perform the exercises. One woman had a mother suffering from problems after pregnancy and said that she felt as though she owed her mother to take advantage of the facilities that were provided for her:

Yes, I think this exercise is for me and I did it. Our mother gave us birth, we are grown up now. During that time, she didn`t get proper facilities like now and now she has different problems and thinking about this, I regularly perform [exercise] out of fear in order to prevent these problems like her and also I enjoyed performing it.

Woman 4.

As a result of having understood the beneficial qualities of the PFMT, several of the women talked about wanting to teach friends and relatives how to perform the exercises as well; not only sharing with other pregnant women, but also teaching older women the exercises as a preventative measure.

Two central and positive factors expressed by the women were that they liked exercising together and being taught together. Exercise in groups gave them an opportunity to ask questions and do the training together with friends. Face to face was mentioned a preferred way of exchanging information. One woman thought that the PFMT-program would be even more beneficial for pregnant women if they were taught by the same health professional each time. Another woman expressed that she thought the program would be more easily understood by educated women compared to women from village areas who, according to her, would have a harder time grasping the full value of the exercise benefits. As a solution, she suggested arranging camps in the remote areas. Yet another woman backed this up by emphasising that the program should be made available to all women, and especially women from rural areas as they are the most exposed to hard physical labour during pregnancy.

The general opinion toward the usefulness of the pedagogic tools agreed on that the video was good, making it easier to understand the different PFMT positions. The video also contributed to emphasizing the importance of the exercises. One woman talked about watching the video with her sister and afterwards the sister recommended her to perform the exercises. Opinions on the usefulness of the leaflet differed. Some did not find it particularly useful having understood how to perform the exercises, while others, particularly those who were unsure how to perform correctly without visual instructions, appreciated the use of pictures related to exercise performance.

I could do that by looking at the leaflet.

Woman 5.

Yes, I was given but I remembered myself; so I didn`t need on daily basis … I had already understood, so I didn`t use it [the leaflet] much.

Woman 4

The general reaction toward the use of an intravaginal biofeedback tool (i.e. electromyography (EMG) registration of PFM activity) for visualization and feedback on correct PFM contraction was less appreciated. Not all the women understood why the device was necessary to use as they did not comprehend the signals displayed on the monitor. Some expressed feeling shy when trying out the tool. A mixed reaction was expressed toward documenting their training performance in an individual diary and was described as not necessary.

There was strong agreement in the interviews that receiving compliments and other optimistic comments on their exercise performance was positive. Being told that they had performed well or done something correctly seemed to increase the women’s self-esteem and motivation for PFMT. One woman talked about a follow-up at the hospital for a perineal tear during childbirth. The doctor in the postnatal ward had been so impressed by her wound healing that he wanted to show her off to all the patients in the ward to encourage them to keep a strong a pelvic floor. The doctor`s words made her feel better about herself and she expressed no longer feeling sick because of the tear.

At the end of the interview the women were asked whether they had anything they would like to add that had not been talked about, but no one volunteered any further information or comments.

## Discussion

The key results of this study indicate that the PFMT-program was well received. The interviewed women all reported they had learned and understood the importance of PFMT and that they performed the exercises daily during pregnancy despite a busy life. The instructional video, conversations with health professionals and individualized group training were perceived as helpful. Support from family and health personnel was important for compliance. In all, the PFMT program appeared successfully adapted into the Nepali context.

The interviews gave the impression that the women were interested in self-care and understood the benefits of performing PFMT during pregnancy. Before attending the PFMT-program, the women had heard about UP and UI, and risk factors, but did not know anything about the PFMs. From the program they said they had learned about the PFMs, how to do the exercises and that those would help to prevent the risk or decrease severity of UP and UI. Among the pedagogic tools the instructional video was considered helpful by all while the leaflets were considered helpful for some to remember the exercises by looking at the illustrations. The individual diary was not emphasised and feedback using the probe did not give any added value. Positive factors were group activity, sharing with other women, and face to face information from health personnel. As a point of improvement was mentioned that one and the same person should conduct all the classes for the group. For compliance doing the exercises, encouragement and understanding from husband or female family members was important.

Even though the PFMT-program was originated from the West, it appeared successfully adapted to Nepali context and the content seemed well received and cultural issues were not brought up as problematic.

Although the sub-sample was representative for the women that participated in the PFMT-program, one should keep in mind that over 90% were living in an urban community and that almost 90% had 12 years of school and that 30% of those had a Bachelor’s degree or higher. This stands in sharp contrast to the reality for many women in Nepal. The average literacy rate in Nepal for females aged 15 years and above was 60% in 2019 [39]. While it was over 75% in the urban areas, female literacy in the rural parts was below 50%. There are also differences between areas where the mountain region has the lowest literacy rate and the hilly central region the highest [40], which is where the Kavrepalanchok district is located and where the Dhulikhel hospital is situated which was where the PFMT-program was conducted. The strategic sample was furthermore mainly represented by women from the highest ethnic castes. The above mentioned factors representing the women in the strategic sample [37] and the sub-sample in the present study: high level of education, high ethnic caste, and living in urban settlement, are all found to be associated with knowledge and awareness of UP. Furthermore, geographical area of residence is also associated with having a satisfactory level of knowledge where central and eastern parts are where the highest percentage of women with a satisfactory level of knowledge about UP are found [41], which is also the region of residence for the women who participated in the PFMT-program [37]. This is also a region with greater access to information through radio, television, and newspapers [41]. The women in the present study were thus well prepared and situated to receive and assimilate the knowledge gained from the PFMT-program.

A study exploring the level of knowledge on UP among married women of reproductive age in Nepal [41] found that less than 50% of the participating women had ever heard of UP, and of those women, only 37.5% knew enough to be categorized as having a satisfactory level of knowledge about the condition [41]. Even fewer seem to recognize what PFMs are [42], which was also true for the women in the present study before attending the program. Good knowledge of these muscles does however not necessarily guarantee that the prescribed exercise is performed [43]. Awareness of potential UP and UI with pregnancy rather than knowledge about PFMs, as told by the women in our study, is not surprisingly more motivating to do the exercises. With the high prevalence and severity reported in Nepal, these Nepalese women may comply no worse with the PFMT regime than Western women where these conditions are (in general) medically well managed. Another motivating factor for Nepali and Western women alike is the experience of symptoms or the fear of developing symptoms of UP [44]. Then again, not all women recognize the symptoms [13] and it is particularly challenging for Nepalese women to seek health care due to fear of stigma. They admit to often avoid the problem because they are too embarrassed to share [45]. The United Nations Population Fund, UNFPA, report that the mean duration for women of suffering from POP in rural Nepal is 7.8 years [46].

Thus, a PFMT-program offered at the antenatal clinic seems as a feasible way to inform women about risk factors, teach the exercises, and motivate the women to do the exercises. The present study indicates that this has been successful. The women in our study performed the exercises because they knew it would benefit them. Even though they had a busy schedule, they somehow found time to perform the exercise and found positions they could fit into their activities, for example while sitting down cooking. They had understood the information provided by the PFMT-program through the different motivational tools and conversations with health professionals who had helped them realize the importance of doing the exercises. The support from female family members or husband was mentioned by the women as motivational and helpful to comply with the program. The family often consists of the husband’s in-laws, and recognition of the suffering by the mother-in-law may lend support [27]. Even though the women mentioned a supportive husband, the husbands’ or men’s knowledge and involvement in female reproductive health is low (also) in Nepal [27]. The PFMT-program at Dhulikhel hospital has taken this to heart by running an informative video in the waiting area at the antenatal clinic. This requires of course that the woman brings her husband. There are also promising reports that couple-friendly reproductive health centers have been both feasible and well accepted [47], despite female disorders being a sensitive subject in Nepal. People in the Nepali culture are generally not used to talking about such topics as shown in a study from 2014 [41], where Nepalese women said they were hesitant to share problems related to reproductive health with family members or others in fear of being embarrassed or tormented. Expressing support and being protective over the wife has furthermore traditionally not been viewed as acceptable for a husband in the Nepali culture. Husbands were afraid of experiencing stigma and were shameful for defying the cultural norms, giving the husband a negative value if acting supportive towards the wife. This seem however being about to change with modernizing of the Nepali society [48].

However, one needs to recognize that there are several contextual factors that are at least as important as exercises. Firstly, the women are small, the average height about 150 cm [19], but the average birth weight is around 3000 g [20]. Second, everyday chores may make it difficult to avoid risk factors, particularly if the family does not recognize or accept that the woman should avoid heavy hard work toward the end of pregnancy and the first period after delivery. If the woman is the youngest, she may be extra vulnerable if living in an extended family where she typically has moved into the husband’s household [49]. Woodsmoke indoors from using a so called *chulo* or *ageno* for cooking and heating wintertime as the houses do not always have chimneys, is causing oxidative stress on the tissues [18] and causes incessant coughing putting strain on the pelvic floor [16]. Increasing deforestation has furthermore forced the women to carry firewood of loads of about 30 kg, often a two hour walk, and to use poorer quality wood and other material making the smoke even worse [50] and adding to heavy labor. Finally, only 57% are giving birth in a health facility [51], a privilege only for those living close enough to a hospital and who can afford it, characterized by high income, high education, and high ethnic caste families [52]. Antenatal services have however now become available also in the rural areas at all public health facilities, including community level primary health-care outreach centers [53]. The government has since 2009 offered a program for free maternity care and transport incentives to promote antenatal care services and institutional delivery [54]. The same factors as mentioned above on economic status, education, ethnic caste, geographical area, and family situation are however still decisive for attending the offered services. Furthermore, beliefs about spirits may prevent women to admit to being pregnant. These factors all contribute to inequalities in women’s health [55].

The answers given by the informants were generally sparse and no one volunteered further information or comments beyond the interviewer’s questions. As mentioned above, women are hesitant to talk about matters concerning female health [56]. Another factor could be an effect of the hierarchical society and an expectation of having to give the ‘correct’ responses to the questions in combination with a portion of shyness. In Nepal, it is considered rude to give negative responses and a person may instead appear positive or remain quiet, thus an indirect mode of conversation is considered polite [57]. More information might have been gained from group interviews instead of one-to-one, where the women could have felt camaraderie as was hinted from the experience with the group-training in the PFMT-program where the women described positive experience from talking to each other.

## Considerations and limitations

Our ambition has been to make the study as transparent and nuanced as possible by describing all steps and choices made to make the study coherent and credible. Due to station, the women may however have kept some details silent. If so, we may still assume that what was told by the women is valid and if something was kept silent, their situation is not exaggerated. Asking the women at the end of the interview whether they would like to add anything that had not been talked about, ensured that they had volunteered all the information that they wanted to share. A strength with the study was that the same person who conducted the interviews in Nepali also made the transcriptions and translations to English, and that this person was a native of the Kavrepalanchok district (4^th^ author). A limitation was however that the interpretation of the material made by the Norwegian team was secondary from the translated transcripts. Thus, non-verbal communication, cultural and linguistic nuances in the ‘inter-view’ [35] between interviewer and the women, might have been lost. Still, the transcripts gave enough information about the participating women’s experiences of performing the PFMT-program they attended during pregnancy, what motivated performance and solutions or potential barriers for compliance. We want to stress that this was, and still is, the best alternative to make the participating women’s voices heard. Consequently, we find the study’s confirmability satisfactory. As in all qualitative studies, our results cannot be statistically generalized to other groups of women, but through our thorough way of reporting, the transferability to other groups of women may be relevant in a pragmatic manner. Note that the women were interviewed about the experience with the PFMT-program during pregnancy. They were not asked about the time postpartum. Still some hinted that they stopped doing the exercises after delivery. It is however advised to continue with the program also after giving birth, c.f [58].

## Conclusion

The present study showed that the PFMT-program was well received and understood. The women had learned about risk factors, the PFMs, how to perform the exercises, and why the exercise was important. They were also eager to share their knowledge. The Nepalese women appear interested in self-care and are making an effort to fit the exercises into their busy schedule. The interviewed sub-sample represented the women who participated in the PFMT-program being rather well educated, of high station, and good economic status, living in an urban or suburban area in the better situated parts of Nepal. This is far from representative for many women in Nepal. For equal rights to health care, the PFMT-program needs to be extended to the antenatal care services at the community out-reach centers. This needs to be followed by another feasibility study on implementation. Keeping up the exercises in the postpartum period should be emphasized. In addition, the situation for many women needs to be improved regarding human rights, equality, and integrity.

## Paper context

Pelvic floor disorders are common among Nepalese women. To prevent or reduce the risk of pelvic organ prolapse and incontinence, a novel pelvic floor muscle training program for pregnant women was introduced. Pelvic floor exercises have proven effective and are recommended internationally. Interviews with the participants revealed that they understood and complied with the program and that self-care was important. We recommend implementation of the program into standard maternity care in Nepal.
